# A degeneration-reducing criterion for optimal digital mapping of genetic codes

**DOI:** 10.1016/j.csbj.2019.03.007

**Published:** 2019-03-19

**Authors:** Helena Skutkova, Denisa Maderankova, Karel Sedlar, Robin Jugas, Martin Vitek

**Affiliations:** Department of Biomedical Engineering, Brno University of Technology, Technicka 12, 616 00 Brno, Czech republic

## Abstract

Bioinformatics may seem to be a scientific field processing primarily large string datasets, as nucleotides and amino acids are represented with dedicated characters. On the other hand, many computational tasks that bioinformatics challenges are mathematical problems understandable as operations with digits. In fact, many computational tasks are solved this way in the background. One of the most widely used digital representations is mapping of nucleotides and amino acids with integers 0–3 and 0–20, respectively. The limitation of this mapping occurs when the digital signal of nucleotides has to be translated into a digital signal of amino acids as the genetic code is degenerated. This causes non-monotonies in a mapping function. Although map for reducing this undesirable effect has already been proposed, it is defined theoretically and for standard genetic codes only. In this study, we derived a novel optimal criterion for reducing the influence of degeneration by utilizing a large dataset of real sequences with various genetic codes. As a result, we proposed a new robust global optimal map suitable for any genetic code as well as specialized optimal maps for particular genetic codes.

## Highlights

• Optimization of 1D numerical representation for DNA to protein translation.
• Reducing genetic code degeneracy in numerical representation of DNA sequences.
• More robust numerical conversion used for genomic-proteomic analysis.

## Graphical Abstract

**Figure:**
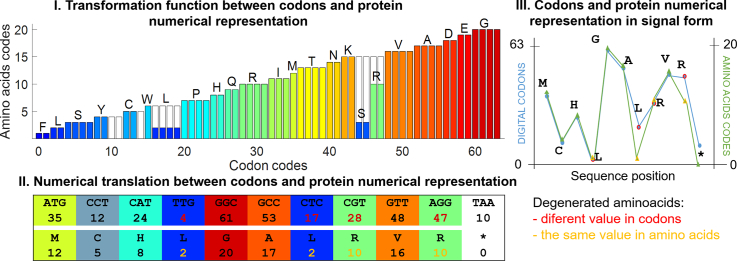

## 1 Introduction

Along with the development of an alternative bioinformatics field referred to as ‘genomic signal processing’ [1], numerical representations of biological sequences have become quite popular. One of the first numerical representations for DNA sequences was the H-curve [2], proposed in 1983. However, there has been a real boom in this field in the last 20 years. Representations which allow visualization of the sequence characteristic trend as a curve in two- or three-dimensional space predominate [[3], [4], [5], [6], [7]], but 4D [8] and 5D [9] representations are also used. With the exception of dimensionality, the numerical representations can be sorted according to their level of degeneration of genetic information, which is caused by conversion from a symbolic representation to the numerical representation [10]. Furthermore, there are representations suitable for DNA and RNA [11], codons [3,5,12], and protein [[13], [14], [15]] sequences. In addition, many numerical representations utilize only a part of the genetic or biochemical information carried by the sequence [12,[16], [17], [18], [19]] and thus their classification is ambiguous. In general, the standardization of numerical representations requirements is missing, e.g. information content, redundancy, convertibility, interpretation etc. Despite that, the analyses based on numerically represented sequences are now well accepted in bioinformatics as described below. Processing of DNA sequences represented as signals is applicable to many problems where symbolic sequences are used, e.g. sequence alignment [20], phylogenetic analysis [21,22], or localization of replication origin [23,24]. Moreover, there are signal-based tools enabling types of analyses that are impossible or highly ineffective for symbolic sequences, especially analysis of periodic features [[25], [26], [27], [28], [29], [30]]. Although papers attempting to systematize methods of numerical representations [1,[31], [32], [33], [34]] or normalize methods of conversion of biological sequences [32,35,36] have been published, a conversion between most of the numerical representations is not possible. Therefore, it is difficult to compare results between analyses employing different numerical representations. Moreover, many numerical representations are designed only for a particular analysis and are never used again.

Instead of proposing another specific numerical representation, we present an optimization that allows more precise and wider use of known numerical representation. A very simple 1D numerical representation of nucleotides by integers (specifically 0, 1, 2, 3) was chosen for optimization in a way preserving whole genetic information and allowing the conversion of nucleotides to codons and then numerical translation to amino acids. The numerical translation results in the loss of information, caused by degeneration of the genetic code as well as translation in symbolic form. The goal of the optimization is to minimalize the information loss by using the ideal permutation of integers, referred to as the numerical map, assigned to sequence residues. The only published optimal map [32] using permutation *T* = 0, C = 1, A = 2, and G = 3 can be further refined for analysis of real sequences as our work shows. The map was proposed only for the standard genetic code and its utilization with other codes is therefore problematic. Our optimization, based on more biologically relevant criteria, chooses the ideal numerical permutation for 24 known genetic codes according to the National Center for Biotechnology Information (NCBI) and forms one universal numerical map. The map is verified using real sequences. Although it is a very simple numerical map, based on four real numbers, its application is wide, starting from simple indexing residues in matrices [33] to complex spectral analysis [28]. The simplicity allows to speed-up calculations. Minimizing the influence of the genetic code degeneracy will allow more effective connection in genomic-proteomic analysis. The preservation of the similarity between genomic and proteomic signals after translation makes possible searching genes in whole genome sequences based on protein query of closely-related species. There is no whole genome translation requirement as is the case of BLAST modification (basic local alignment search tool) – tblastn [37,38]. This enables e.g. better detection of phenotypically related bacteria based on their expressed protein content or more effective searching of conserved genes, orthologue genes and pseudogenes. This leads to more accurate estimation of core or pan genome for diversification of closely-related pathogenic bacteria [[39], [40], [41]].

## 2 Materials and Methods

### 2.1 The Genetic Codes and Data Used

The hitherto used optimal numerical map (T = 0, C = 1, A = 2, and G = 3) [32] was constructed for the standard genetic code, which is relevant for the majority of linear DNA of eukaryotic organisms. However, the number of genome records utilizing alternative genetic codes is rapidly increasing in public databases as genome sequencing has become an affordable and widely used technique in molecular biology. Therefore, the genomic signal processing should take into account properties of particular genetic codes when new numerical representations are proposed. Although the differences in codon translations are in most cases minimal, an inappropriately chosen numerical map can significantly influence subsequent analyses. Here, we extended the definition of the optimal numerical map by considering all genetic codes. We used the latest systematics of genetic codes (last update: 7 January 2019) according to the NCBI [42]. This systematics is based mainly on reviews by Jukes and Osawa [43] and Osawa et al. [44]. In the last year, it was extended by codes 24–31. On the other hand, seven code numbers were eliminated (numbers 7, 8, 15, 17, 18, 19 and 20). These genetic codes were updated (e.g. new taxonomy classification) and obtained a new higher number. All up-to-date 24 genetic codes are listed in Table 1.

**Table 1:** A summary of all genetic codes.

Code Number	Code Name
1	Standard
2	Vertebrate Mitochondrial
3	Yeast Mitochondrial
4	Mold, Protozoan, and Coelenterate Mitochondrial
5	Invertebrate Mitochondrial Code
6	Ciliate, Dasycladacean and Hexamita Nuclear
9	Echinoderm and Flatworm Mitochondrial
10	Euplotid Nuclear
11	Bacterial, Archaeal and Plant Plastid
12	Alternative Yeast Nuclear
13	Ascidian Mitochondrial
14	Alternative Flatworm
16	Chlorophycean Mitochondrial
21	Trematode Mitochondrial
22	*Scenedesmus obliquus* Mitochondrial
23	Thraustochytrium Mitochondrial
24	Pterobranchia Mitochondrial
25	Candidate Division SR1 and Gracilibacteria
26	Pachysolen tannophilus Nuclear
27	Karyorelict Nuclear
28	Condylostoma Nuclear
29	Mesodinium Nuclear
30	Peritrich Nuclear
31	Blastocrithidia Nuclear

In this paper, we report the construction of the optimal numerical maps for genetic codes for which translational tables are available on the afore mentioned NCBI website. Basic translational tables were used; no special cases were incorporated. The theoretically derived numerical maps were verified using real sequences. A verification dataset was created for selected genetic codes. Each dataset was comprised of DNA sequences for 50 genes from several organisms. The genetic codes for which only few sequences, mostly from one species, are available in databases were excluded from our study as their optimal numerical maps cannot be reliably verified. Only records of sequences containing a note of used translational tables were added to the datasets. There was also a condition that the records annotations must be verified (not only automatically annotated or predicted) and must include CDS location, because identification of mRNA segments is not enough. In total, datasets covering 13 different genetic codes were used to verify the proposed versatile numerical map. A summary of the used sequences in datasets for each of the 13 genetic codes is shown in Table 2.

**Table 2:** Numbers of sequences in datasets for each of the 13 genetic codes.

Organism	Number of sequences	Genetic code	Source	Organism	Number of sequences	Genetic code	Source
*Homo sapiens*	15	1	nuc	*Oxytricha nova*	6	6	nuc
*Pongo abelii*	15	1	nuc	*Paramecium tetraurelia*	25	6	nuc
*Pan troglodytes*	20	1	nuc	*Stylonychia lemnaepartial*	3	6	nuc
*Pan troglodytes ellioti*	13	2	mt	*Tetrahymena thermophila*	3	6	nuc
*Homo sapiens*	12	2	mt	*Gyrodactylus brachymystacis*	12	9	mt
*Gorilla gorilla*	13	2	mt	*Paragonimus ohirai*	12	9	mt
*Pongo abelii*	12	2	mt	*Fasciola jacksoni*	12	9	mt
*Saccharomyces cerevisiae*	8	3	mt	*Microstomum lineare*	5	9	mt
*Candida glabrata*	9	3	mt	*Taenia asiatica*	9	9	mt
*Kluyveromyces thermotolerans*	11	3	mt	*Euplotes nobilii*	8	10	nuc
*Eremothecium sinecaudum*	7	3	mt	*Euplotes raikovi*	2	10	nuc
*Lachancea kluyveri*	7	3	mt	*Euplotes charon*	1	10	nuc
*Saccharomyces pastorianus*	8	3	mt	*Euplotes focardii*	2	10	nuc
*Tetrahymena pyriformis*	18	4	mt	*Euplotes vannus*	17	10	nuc
*Leishmania tarentolae*	4	4	mt	*Euplotes octocarinatus*	20	10	nuc
*Plasmodium gallinaceum*	3	4	mt	*Escherichia coli*	33	11	nuc
*Chondrus crispus*	13	4	mt	*Mycobacterium tuberculosis*	17	11	nuc
*Choreocolax polysiphoniae*	7	4	mt	*Candida dubliniensis*	30	12	nuc
*Kappaphycus striatus*	5	4	mt	*Candida albicans*	20	12	nuc
*Caenorhabditis elegans*	12	5	mt	*Halocynthia roretzi*	12	13	mt
*Caenorhabditis briggsae*	12	5	mt	*Ciona savignyi*	12	13	mt
*Ascaris suum*	12	5	mt	*Clavelina phlegraea*	13	13	mt
*Ascaris lumbricoides*	12	5	mt	*Ascidiella aspersa*	13	13	mt
*Katharina tunicata*	2	5	mt	*Pediastrum duplex*	12	22	mt
*Euplotes petzi*	6	6	nuc	*Scenedesmus obliquus*	20	22	mt
*Acetabularia cliftonii*	3	6	nuc	*Tetradesmus obliquus*	18	22	mt
*Acetabularia acetabulum*	3	6	nuc	*Candidate division*	30	25	nuc
*Acetabularia peniculus*	1	6	nuc	*Candidatus Gracilibacteria*	20	25	nuc

### 2.2 The Principle of Numerical Conversion

The construction of the optimal numerical map begins with a simple 1D numerical map for DNA sequences, which assigns a scalar value to each nucleotide. A numerical vector is obtained by sequential replacement of nucleotide symbols with their scalar representatives. The resulting vector has the same length as the original symbolic sequence. A number of 1D numerical maps for nucleotides using real numbers, which represent some of the physical or biochemical features of the nucleobases, can be found. For example, numerical map A = 0.1260, C = 0.1340, G = 0.0806, and *T* = 0.1335, representing EIIP values of the bases [45], while map A = 70, C = 58, G = 78, and *T* = 66, representing atomic numbers of the bases [46]. Another possibility is to highlight the complementarity of the bases A = −1.5, *T* = 1.5, C = 0.5, and G = −0.5 [47] or the general occurrence of purine/pyrimidine bases A or G = −1, C or *T* = 1 as a so-called DNA walk [24,48].

Some simple mathematical operations applied on these specific 1D numerical maps, e.g. cumulative sum along the numerical vector, can reveal a specific trend in the sequence [49], but its general utilization is limited. Thus, finding a linkage between the DNA sequence and its translation to protein in their numerical representations is not straightforward.

The basic numerical map consisting of integers {0, 1, 2, 3}, which we chose to optimize, carries the full information content, as does the original symbolic sequence, and can be transformed into any other numerical representation, which is not possible for every numerical representation, e.g. the DNA walk mentioned above. An advantage of the map lies in a simple conversion to codon representation and following determination of the numerical map for translation into amino acids numerical representation. As integers from interval 0–3 are used to represent nucleotides, similarly integers 0–63 and 0–20 are used for codons and amino acids, respectively.

The codon representation is derived directly from the nucleotide representation. One codon is coded by three nucleotides and in the numerical form it corresponds to a three-digit number of the quaternary numeral system. The transformation lies in the conversion of quaternary to decimal numbers. For example, amino acid methionine, which is coded as ATG according to the standard genetic code, has the numerical representation ATG = 203 in the quaternary system defined by the numerical map *T* = 0, C = 1, A = 2, and G = 3. The corresponding decimal number is ATG = 203_4_ = 35_10_. All triplets in the DNA sequence are transformed to the codon representation in this manner. Therefore, a resulting vector of values from interval 0–63 obtained during this simple transformation has one-third the length of the original sequence.

On the contrary, the following conversion to the amino acid numerical representation is not so trivial and the method of conversion has no simple mathematical explanation. The reason can be found in degeneration of the genetic code, caused by 64 codons coding only 20 proteinogenic amino acids. The process of numerical translation was defined by Cristea [32]. Numerical translation begins with the lowest value of the codon numerical representatives TTT = 000_4,_ as defined according to the previously presented numerical map. The standard genetic code translates codon TTT into phenylalanine. Therefore, phenylalanine is assigned the numerical value Phe = 1. The next codon TTC = 001_4_ also translates into phenylalanine. Because phenylalanine has already assigned a numerical representative, the process continues with another codon which is TTA = 002_4_ for leucine. Therefore, the numerical representative is Leu = 2. The next five codons translating into leucine are skipped as the numerical representative is assigned according to the first codon for the same amino acid. This procedure creates a numerical map for all 20 standard proteinogenic amino acids.

**Fig. 1:**
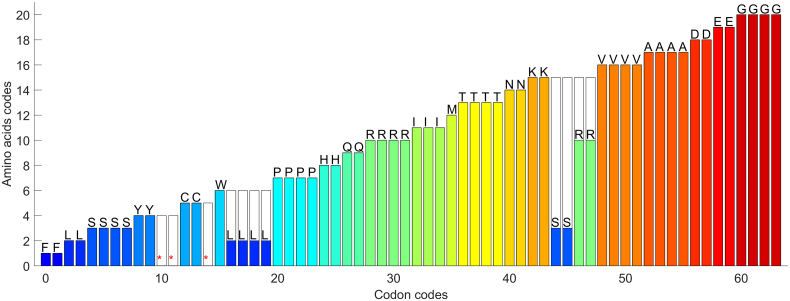
Transformation function between decimal values of codons to decimal values of amino acids, using numerical map for nucleotides *T* = 0, C = 1, A = 2, and G = 3. Each amino acid is assigned an integer from 1 to 20, while 0 values are reserved for terminators.

The amino acids reach values from 1 to 20. Value 0 is reserved for termination codons regardless of the order of the corresponding codon numerical representative. This assignment to termination codons prevents discontinuity in assignments to amino acids. The termination codon is the last codon of a gene sequence and in most sequence analyses it is not used. After the assignment of decimal values to all amino acids, the transformational function can be visualized as depicted in Fig. 1.

### 2.3 The Optimization of Numerical Conversion

There are 24 (factorial 4) possible variants of the assignment when converting the symbolic representation of nucleotides to the numerical representation using integers 0, 1, 2, and 3. The selection of a particular variant of representation is not that important for simple purposes such as indexing. On the other hand, for a complex analysis requiring the conversion of gene CDS to codons and amino acids, the preservation of maximum similarity of the signals in each step of the conversion is highly desirable. The goal of the numerical map optimization therefore lies in the maximization of the signal similarity. The afore mentioned numerical map *T* = 0, C = 1, A = 2, and G = 3 was designed as the optimal numerical map for the standard genetic code based on a simple optimizing criterion, which is the smallest number of degenerated segments *N* in the transformation function. As Fig. 1 shows, this map has *N* = 3 degenerated segments if termination codons are not considered. The degenerated segments are caused by three amino acids (leucine, serine, and arginine), which suffer from degenerescency six. Analyses of real sequences showed that the simple criterion based on the number of degenerated segments was not sufficient for the following reason.

Let's assume that the genetic code is not degenerated or that the level of degeneration is the same for all amino acids. The trend of amino acid signal would then be identical with the codon signal but three times smaller. However, there are different levels of degeneration in the genetic code and this has to be taken into account when optimizing the numerical map. The optimal numerical map provides the highest similarity between signals for codons and amino acids. Higher similarity can be obtained by a more sophisticated optimization criterion.

The original optimization criterion counts only the number of degenerated segments. Here, we propose also to consider the weight of degeneration. The weight of degeneration is determined by the number of codons causing the degeneration and by the drop of transformation function during the degeneration. The weight of degeneration is depicted in Fig. 1 as white columns in the positions of the degenerated segments. The new optimization criterion is based on the length and height of the degenerated segments. For example, the degenerated segment for leucine (see Fig. 1) has length *L* = 4 and height *H* = 4, and the degenerated segment for arginine has *L* = 2 and *H* = 5. The weight of the degenerated segment for leucine is *w*_L_ = *L* x *H* = 4 × 4 = 16, for arginine *w*_R_ = *L* x *H* = 2 × 5 = 10 and for serine *w*_S_ = *L* x *H* = 2 × 12 = 24. The optimization criterion *W* is the sum of all degeneration weights except the termination codons. The example in Fig. 1 has *W* = *w*_L_ + *w*_S_ + *w*_R_ = 16 + 24 + 10 = 50.

The optimal numerical map has the lowest value of optimization criterion *W* from all 24 possible variants. This optimization ensures the minimal divergence between numerical signals for codons and amino acids; therefore, the signals representing real DNA and protein sequences are as similar as possible. Based on this new criterion, the optimal numerical map was derived for each of the 24 genetic codes. Table 3 shows values of the optimization criterion *W* for the resulting optimal numerical maps for all genetic codes. For comparison, the table also shows optimization criterion value *W* for the original numerical map *T* = 0, C = 1, A = 2, and G = 3 and the number of degenerations *N* for all optimal numerical maps. It is evident that the original numerical map has a lower or identical number of degenerations for all genetic codes, but has a higher *W* value in all cases in comparison to the new optimal numerical maps. The previously published map minimized only the number of changes in the signals. Our criterion minimizes also their size. Therefore, it can be assumed that the new optimal numerical maps will cause a smaller dissimilarity between signals for codons and amino acids than the original numerical map.

**Table 3:** The optimal numerical maps and their values of optimization criterion.

Genetic code	Proposed optimal numerical map						Original optimal numerical map [ACGT] = [2 1 3 0]		Globally optimal numerical map [ACGT] = [1 0 3 2]	
	[A	C	G	T]	*W*	*N*	*W*	*N*	*W*	*N*
1	**1**	**0**	**3**	**2**	33	3	50	3		
2	3	1	0	2	20	4	40	2	23	3
3	**1**	**0**	**3**	**2**	20	3	56	3		
4	**1**	**0**	**3**	**2**	34	4	50	3		
5	**1**	**0**	**3**	**2**	23	3	64	2		
6	3	2	0	1	45	4	62	4	51	4
9	**1**	**0**	**3**	**2**	21	3	64	2		
10	**1**	**0**	**3**	**2**	33	4	50	3		
11	**1**	**0**	**3**	**2**	33	4	50	3		
12	3	2	0	1	34	4	49	4		
13	3	0	2	1	44	4	56	3	62	4
14	**1**	**0**	**3**	**2**	21	3	64	2		
16	3	2	0	1	38	5	52	4	41	5
21	**1**	**0**	**3**	**2**	22	3	64	2		
22	3	2	0	1	38	4	52	4	41	5
23	**1**	**0**	**3**	**2**	24	4	50	3		
24	**1**	**0**	**3**	**2**	25	4	52	2		
25	3	2	0	1	42	4	112	4	54	5
26	3	2	0	1	41	4	88	4	79	5
27	3	2	0	1	46	4	62	4	52	4
28	3	2	0	1	46	5	62	4	52	4
29	**1**	**0**	**3**	**2**	33	3	50	3		
30	**1**	**0**	**3**	**2**	45	4	84	4		
31	**1**	**0**	**3**	**2**	46	5	84	4		

Bold highlighted text indicates where is the global optimal map correspond with local map for particular genetic code.

In addition to the optimal numerical map for each of the known genetic codes, a globally optimal numerical map was derived. The globally optimal map can be used for sequences without a defined genetic code or for applications where the same settings are needed. The global optimum was chosen according to the minimal suitability score of the numerical map variants. The suitability score of each numerical map variant was calculated as follows. Firstly, for each variant of numerical map, *W* values were calculated for all 24 genetic codes. An order of suitability of numerical maps for each genetic code was defined based on *W* values. The suitability score of the given numerical map variant is the sum of its suitability orders for all genetic codes. The suitability score eliminates the effect of very low or high *W* values and ensures that the chosen globally optimal numerical map is not extremely unsuitable for some of the genetic codes. The resulting globally optimal map is defined as A = 1, C = 0, G = 3, and *T* = 2. This map is also the optimal map for 14 of the 24 genetic codes. Table 3 also highlights values *W* and *N* of the globally optimal map for genetic codes having a different optimal numerical map than the global one. In these cases, the globally optimal map has a slightly worse *W* value than the optimal map. Yet, with the exception of genetic code nr. 13, it is better than the original numerical map based only on *N* criterion.

## 3 Results and Discussion

### 3.1 Evaluation of Signal Distortion Caused by Translation

The goal of the proposed optimization was to achieve minimal divergence from a linear trend of the transformation function, which leads to the minimal difference between genomic and proteomic signal. The difference is caused by degeneration of the genetic code. The optimal numerical map can minimalize distortion of the numerical representation of translated protein. We evaluated the influence of the map to signal distortion. Two frequently used parameters for evaluation of the differences between two signals were used: Pearson correlation coefficient (corrcoef) and percentage deviation (*D*). Because the corrcoef parameter is not affected by mean value of the signals it can be used as a quality criterion in its basic definition. On the contrary, the percentage deviation needs adjustment of the signal ranges. Both signals have to be normalized by their maximal possible value, which is 63 for codon signal and 20 for amino acid signal. The percentage deviation *D* for the normalized signals can be computed as

$$D=\frac{\sum_{n=1}^{M}c\left[n\right]/63-a\left[n\right]/20}{M}\times 100,$$

where *c*[*n*] is the codon signal and *a*[*n*] is the amino acid signal of the translated protein. Both signals have the same length *M*, which is the number of codons or amino acids.

**Fig. 2:**
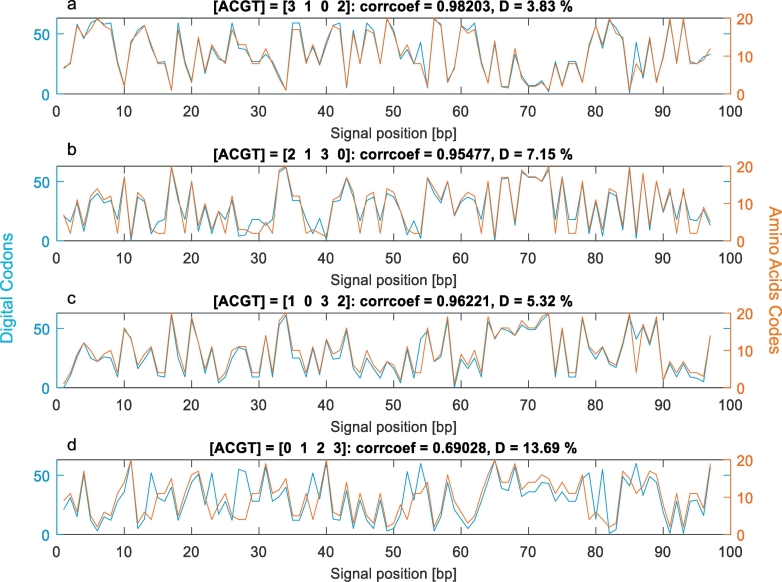
Comparison of codons and amino acids signals for different numerical maps: a) new optimal numerical map for genetic code vertebrate mitochondrial; b) original optimal numerical map; c) new general numerical map; d) randomly chosen numerical map.

Short protein dehydrogenase subunit 4 L (NADH) from the mitochondrial DNA sequence of the common chimpanzee (*Pan troglodytes*, accession number AEQ36160) was used to demonstrate the influence of various maps to signal distortion. The protein was translated from the DNA sequence according to the genetic code nr. 2 – vertebrate mitochondrial. The effect of the numerical map on signal distortion for four different numerical maps is shown in Fig. 2. Fig. 2a) shows the codon signal and the amino acid signal when the optimal numerical map for genetic code nr. 2 was used. The map is A = 3, C = 1, G = 0, and *T* = 2. It is evident that both signals are very similar. Their percentage deviation is less than 4% and corrcoef is over 0.98, which shows very high mutual dependence of both signals. Subplot 2b) shows both signals for the original optimal map A = 2, C = 1, G = 3, and *T* = 0, which was proposed in [32], and subplot 2c) shows both signals for our globally optimal map A = 1, C = 0, G = 3, and *T* = 2. These two numerical maps produced signals with a slightly higher dissimilarity than the optimal numerical map for the given genetic code. Nonetheless, both maps preserved a high level of similarity with percentage deviation under 10% and correlation coefficient over 0.95. It is notable that our globally optimal map gave better results than previously published optimal map. For comparison, subplot 2d) shows signals for a randomly chosen numerical map that was not optimal for any of the genetic codes. As parameter *D* indicates, the non-optimal map caused four times higher distortion than the optimal map. The correlation parameter under 0.7 signifies moderate dependence of the signals.

We evaluated signal distortion for the optimal numerical maps of 13 genetic codes. Each genetic code was represented by 50 real DNA sequences. As the results differ for each sequence and genetic code, a mean corrcoef and its standard deviation (STD) were calculated. The results are summarized in Table 4. Similarly, a mean percentage deviation was evaluated, as Table 5 shows. In both tables, the first column corresponds to the genetic code used, the second column summarizes the results for our optimal numerical maps, the third column is for previously published optimal map, and the fourth column shows the results for our globally optimal map in cases where it is not identical to the proposed optimal map for a specific genetic code. The last two columns show the best and the worst results from all other variants of numerical map. The best results are shown only in cases where they differ from the results of the proposed optimal map. Our optimal numerical maps are not always the best possible maps for real sequences (e.g. genetic code 2) because these maps were theoretically derived with the assumption of uniform codons distribution in sequences. Cases where this theoretical assumption is not satisfied are discussed below.

**Table 4:** Evaluation of genomic signal distortion based on Pearson correlation coefficient.

Genetic code	Optimal numerical maps for genetic codes	Original optimal numerical map [ACGT] = [2 1 3 0]	Globally optimal numerical map [ACGT] = [1 0 3 2]	Best result	Worst result
1	0.9647 ± 0.0273	0.9168 ± 0.0173			0.6224 ± 0.0901
2	0.9277 ± 0.0574	0.9180 ± 0.0770	0.9362 ± 0.0762	0.9567 ± 0.0831	0.5604 ± 0.1171
3	0.9544 ± 0.0315	0.9478 ± 0.0235			0.7123 ± 0.0782
4	0.9224 ± 0.0370	0.9210 ± 0.0297			0.4903 ± 0.1844
5	0.9217 ± 0.0443	0.8714 ± 0.0608			0.5195 ± 0.0871
6	0.9547 ± 0.0105	0.8895 ± 0.0532	0.9395 ± 0.0136	0.9689 ± 0.0129	0.6267 ± 0.0423
9	0.9396 ± 0.0194	0.8984 ± 0.0393			0.5761 ± 0.1060
10	0.9518 ± 0.0182	0.9478 ± 0.0197			0.7839 ± 0.0633
11	0.9550 ± 0.0216	0.9400 ± 0.0249			0.6384 ± 0.0883
12	0.9244 ± 0.0454	0.9176 ± 0.0401	0.9105 ± 0.0455	0.9388 ± 0.0348	0.6015 ± 0.0813
13	0.9288 ± 0.0219	0.9029 ± 0.0232	0.8898 ± 0.0339		0.5560 ± 0.1209
22	0.9456 ± 0.0226	0.9121 ± 0.0376	0.8893 ± 0.0670		0.6297 ± 0.1017
25	0.9410 ± 0.0470	0.7905 ± 0.0681	0.8454 ± 0.0763		0.6649 ± 0.0970

**Table 5:** Evaluation of genomic signal distortion based on percentage deviation *D.*

Genetic code	Optimal numerical maps for genetic codes	Original optimal numerical map [ACGT] = [2 1 3 0]	Globally optimal numerical map [ACGT] = [1 0 3 2]	Best result	Worst result
1	4.74 ± 0.88	6.83 ± 0.66			16.52 ± 1.76
2	4.39 ± 1.96	7.12 ± 1.13	5.72 ± 1.27	3.9853 ± 2.0414	16.50 ± 2.57
3	3.47 ± 0.94	7.66 ± 0.95			15.83 ± 1.47
4	7.55 ± 1.33	8.04 ± 2.20			16.58 ± 1.99
5	7.63 ± 1.50	8.93 ± 1.74			14.20 ± 1.24
6	5.38 ± 0.65	8.82 ± 1.91	5.80 ± 0.53		18.53 ± 0.56
9	7.00 ± 0.92	8.03 ± 1.37			13.81 ± 2.53
10	4.93 ± 0.61	5.79 ± 0.65		4.6911 ± 0.6924	13.87 ± 1.03
11	4.04 ± 1.45	5.78 ± 1.04			17.46 ± 1.66
12	6.79 ± 1.66	7.08 ± 1.42	7.45 ± 1.23		16.45 ± 1.61
13	7.72 ± 1.40	8.44 ± 0.95	9.02 ± 1.14		15.72 ± 2.23
22	6.60 ± 0.86	7.53 ± 0.93	8.63 ± 1.40		15.03 ± 1.27
25	6.73 ± 1.89	12.54 ± 1.41	8.63 ± 1.09		16.79 ± 2.53

**Fig. 3:**
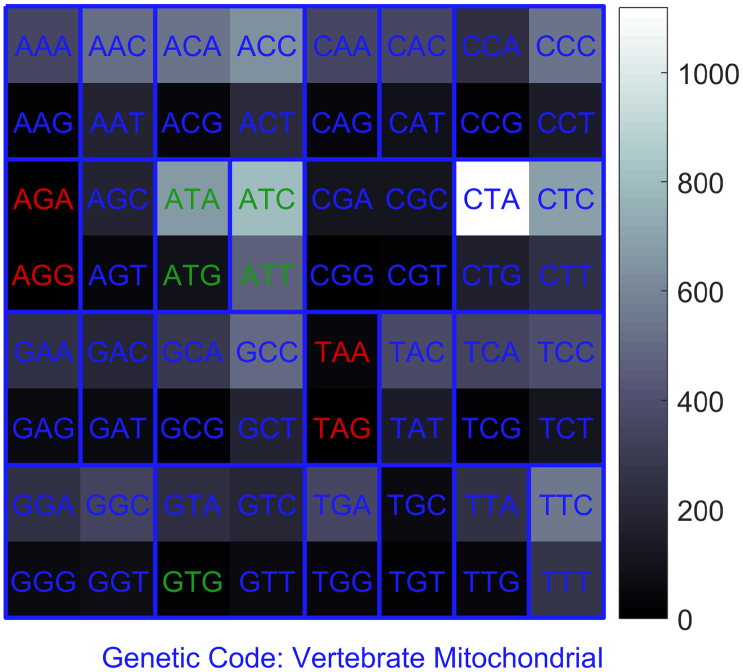
Codon frequencies for 50 vertebrate mitochondrial coding sequences. Termination codons are marked red and initiation codons green. (For interpretation of the references to colour in this figure legend, the reader is referred to the web version of this article.)

The results summarized in Table 4 and 5 suggest that optimization of the numerical map is necessary as the signal distortion can be higher than 20% (considering mean value and standard deviation of *D*). In some extreme cases, the correlation coefficient dropped under 0.5, which denies the assumption that the codon signal and the amino acid signal are closely related. Our optimal maps gave, in most cases, better than or at least comparable results to the original optimal map. There were only three cases when the corrcoef was higher for another variant of numerical map and two cases when the percentage deviation was also lower. This was caused by codon usage bias, which is a different frequency of codons for one amino acid. The codon bias is quite common in bacterial [50] and viral [51] DNA or RNA and is also reported in mitochondrial DNA of vertebrates [52]. For example, our optimal numerical map for vertebrate mitochondrial DNA did not provide the finest results for this genetic code. Its percentage deviation 4.39% was the second best. In this case, the corrcoef was also higher for the globally optimal map. Fig. 3 shows the frequencies of codons in total for 50 vertebrate mitochondrial sequences that we used to evaluate the proposed optimal numerical maps.

Our optimization criterion tries to minimalize the influences of extensive degeneration of amino acids. For example, amino acid leucine is coded by six codons. Four of them are mapped next to each other by the transformational function as they differ only by one nucleotide in the third position within the codon (CTA, CTC, CTG, CTT) that has the smallest informational weight. The remaining two codons (TTA, TTG) differ in the first position. During the optimization, we attempted to minimize the difference in assigned values between these two and the remaining four codons as the difference causes non-monotony (occurrence of degenerated segment) in the transformation function, leading to distortions in the signals. The more codons used to assign a value to the amino acid, the lower the weight of degenerated segment. Therefore, a value for leucine should be assigned according to the four neighboring codons rather than the remaining two. Although the proposed optimization minimalizes these issues, there is a certain loss of signal resolution as several different codons have the same value for amino acid and therefore the amino acid signal differs from the codon signal. If the codon distribution in sequence is uniform, the signal distortion is minimal. On the other hand, if a single overrepresented codon such as CTA in our vertebrate mitochondrial dataset shown in Fig. 3 is present, the signal distortion is noticeable. The codon CTA forms more than one-fifteenth of the sequences (1119 CTA out of all 14,942 codons). The proposed optimal map A = 3, C = 1, G = 0, and T = 2 assigns the value 123_4_ = 27_10_ to the codon CTA, which is three more than assigned to another leucine codon, CTG, with value 120_4_ = 24_10_. The CTG codon had frequency 167 in the dataset. A similar issue applies to codons ACA (freq. 536) and ACG (freq. 36) for the amino acid threonine, and codons TCA (freq. 333) and TCG (freq. 26) for serine. That is the reason why the globally optimal map A = 1, C = 0, G = 3, and *T* = 2 is slightly better than the optimal map as the difference in numerical values for adenine and guanine is only 2.

For these reasons, the proposed optimization criterion was not sufficient and the results strongly depended on features of the particular analyzed sequences. For example, Fig. 2 shows signals for vertebrate mitochondrial sequence for which the optimal numerical map was also the best variant.

In addition, an average value of correlation coefficient exceeded 0.9, which is sufficient for most of the common analyses, e.g. motif searching or comparative analysis. For more precise results, an additional optimization based on features of analyzed sequences such as codon bias is necessary. Many of the genetic codes are newly discovered and thus public repositories lack a sufficient number of sequences for reliable optimization. Frequently, only a single sequence is available. For these insufficiently represented genetic codes, it is convenient to use a numerical map based on a simple, clear and general optimization criterion. Another possibility is to use a globally optimal numerical map independent of the genetic code.

To conclude, the proposed globally optimal map provides better results than the original optimal map in 10 out of 13 cases. In the remaining three cases, the results are slightly worse. There was even a single case in which the original optimal map was significantly worse than our maps. It was the case of genetic code nr. 25 for which the average percentage deviation was above 10% and the correlation coefficient was under 0.8. The globally optimal map was identical to the proposed optimal maps for seven out of 13 tested genetic codes. Additionally, for six of these seven codes, this map gave the best results, with a single exception in genetic code nr. 10, where a map with slightly better percentage deviation could be found.

### 3.2 Phylogenetic Example

**Fig. 4:**
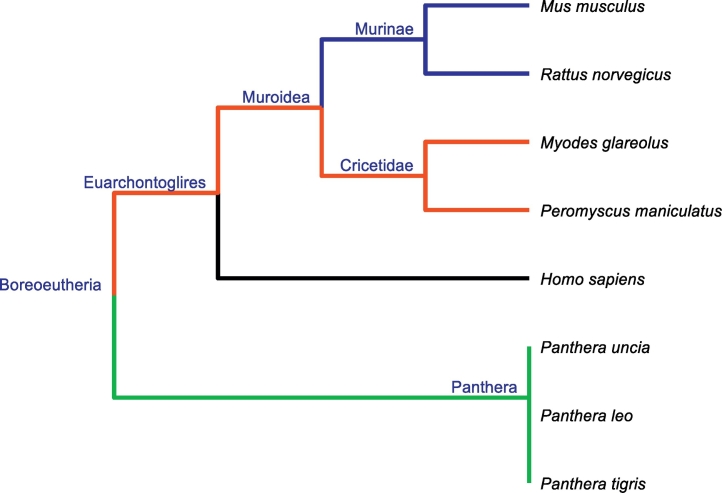
Reference taxonomic tree of tested organisms according to NCBI taxonomy.

In addition to the analysis of signal distortion caused by translation, an influence of signal distortion on the topology of phylogenetic tree was tested. A dataset covering eight protein coding sequences of HBB (beta globin) genes of mammals from GenBank was used; see Table 6. All sequences have the same length of 444 nucleotides, 148 codons/amino acids, respectively. Therefore, no signal alignment was needed. The close phylogenetic relationship of some species allowed us to examine the resolution of phylogenetic classification using the signals. A reference taxonomic tree was constructed according to taxonomy published at NCBI [53,54] and is shown in Fig. 4.

**Table 6:** Overview of tested organisms.

Accession no.	Organism
KR818803	*Panthera leo*
KR818801	*Panthera uncia*
KR818802	*Panthera tigris*
NM_001278161	*Mus musculus*
KJ677213	*Myodes glareolus*
KJ725788	*Peromyscus maniculatus*
M17084	*Rattus norvegicus*
KU350152	*Homo sapiens*

The phylogenetic analysis was conducted on numerical representations of protein sequences, while employing three different numerical maps. These maps were derived from numerical maps for nucleotides, as previously described. A mutual distance of two protein signals was calculated as proportional deviation *d:*

$$d=\frac{\sum_{n=1}^{M}\left|a_{1}\left[n\right]/20-a_{2}\left[n\right]/20\right|}{M}$$

where *a*_1_[*n*] and *a*_2_[*n*] are protein signals of length *M*.

**Fig. 5:**
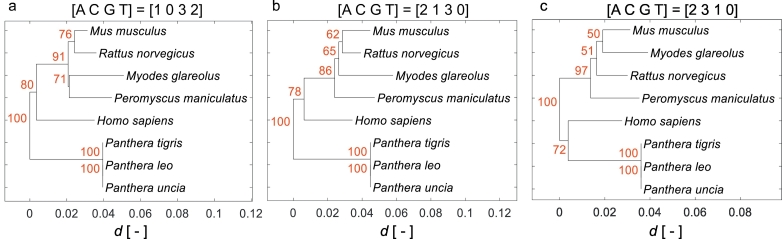
Phylogenetic trees constructed from protein signals based on three numerical maps: a) new optimal numerical map; b) original numerical map; and c) random numerical map. Red labels of branch nodes represent results of statistical verification by bootstrapping test (percentage values from 1000 replications). (For interpretation of the references to colour in this figure legend, the reader is referred to the web version of this article.)

Although the signal normalization was not necessary as both compared signals had the same value range, it was preserved to maintain consistency with the previous evaluation of signal distortion. The proportional deviation was calculated for all pairs of signals to compile the distance matrix, and the phylogenetic tree was constructed using neighbor-joining [55]. The resulting phylogenetic trees, using three different maps for the standard genetic code, are shown in Fig. 5. The first map is the newly proposed optimal map A = 1, C = 0, G = 3, *T* = 2, which is also the globally optimal map for all genetic codes. The second is the original optimal map A = 2, C = 1, G = 3, *T* = 0, and the third is the non-optimal assignment of nucleotides A = 2, C = 3, G = 1, *T* = 0.

The trees of all numerical maps were compared to reference tree by calculating Robinson-Foulds distance (RFdist) [56] for rooted trees using R software (packages phytools and phangorn). The robustness of the phylogenetic trees was evaluated by the bootstrapping statistical test [57]. The implementation of this standard statistical test for symbolic sequence based phylogenetic trees is practically identical for genomic signal based trees. However, it must be taken into account that each mutation in numerical representation have different influence to result tree. The variability of bootstrap replications is therefore much higher than for symbolic sequences. While symbolic sequence based methods need at least 100 bootstrap replications for reliable statistical verification, the genomic signal based implementation requires 1000.

Despite this being a very simple classification task, only our optimal map led to the phylogenetic tree being similar to the reference tree with RFdist equal to 0. The original numerical map caused a split in the Cricetidae family (RFdist = 0.167) and the random numerical map caused disorder in the internal arrangement of the Muroidea superfamily cluster. Moreover, the non-optimal map classified humans as being closer to carnivorans than to rodents (RFdist = 0.5) and caused an overall decrease in proportional deviation. This suggests that the non-optimal numerical map decreases the classification resolution of signal representations. This fact is also confirmed by the robustness of the phylogenetic tree, where the bootstrap supports of nodes in non-optimal map tree are lower than in other two.

## 4 Conclusion

The aim of this paper is to contribute to the standardization of basic operations in genomic signal processing, which is a rapidly developing new branch of bioinformatics. The proposed optimization sets new rules for the first step of genomic signal processing, which is the transformation of symbolic sequences to numerical representation. In comparison with other authors, we are not proposing a new type of sophisticated numerical transformations, which are frequently suitable only for one type of analysis, but we optimize the known conversion of nucleotides to integers 0, 1, 2, and 3. This numerical mapping is simple, versatile and currently widely used. Many users of bioinformatics software are using it unknowingly. Computational functions prefer processing of numbers rather than symbols. This simple numerical map and its variations, based on different assignments of values to nucleotides, can be optimized for the purposes of complex analyses of DNA sequences and proteins, e.g. genome mapping or comparative genomics. For this purpose, it is necessary to minimalize the loss of genetic information caused by translation in the numerical form.

Although the numerical map was already optimized [32], the optimization criterion was set simply as a number of amino acids degenerations and the resulting numerical map is not robust enough for the processing of real data. We proposed optimization according to a new optimization criterion that is focused on minimizing information loss between genomic and proteomic signals. The optimal numerical map ensures maximal similarity of the numerical representation of nucleotides and amino acids despite the degeneration of the genetic code. The basis of optimization criterion lies in minimizing the divergence of numerical values of codons representing multiple degenerated amino acids, e.g. leucine with six codons. This optimization takes into account not only a number of degenerated amino acids but also the weight of introduced errors. Another disadvantage of the original optimal numerical map comes from its exclusive definition only for the standard genetic code. Therefore, the selected variant of value assignment is not optimal for alternative genetic codes and its general utilization is limited.

We applied the new optimization criterion to all known genetic codes to derive particular optimal maps. Moreover, we were able to propose the globally optimal map based on complex analysis of optimization criterion results for all 24 variants of numerical map for each of 24 genetic codes.

The proposed optimal maps for specific genetic codes as well as the globally optimal map were verified using 650 gene sequences of different organisms and different types of DNA, e.g. nuclear, mitochondrial etc. Results of verification were compared to results for the original optimal map and to the worst and the best cases of all numerical maps. Two parameters, correlation coefficient and percentage deviation, were used to evaluate dissimilarity between codon and amino acid signal. While the first of them quantifies dependence between signals before and after translation, the latter evaluates dissimilarity of these signals. For most of the genetic codes, the best results were obtained using our newly proposed optimal maps. Three cases of slightly better results of percentage deviation and only two cases of correlation coefficient were recorded when using other maps. In these cases, the results depended heavily on sequences used for verification due to their codon bias, which manifests differently for various organisms. An additional optimization is needed for more precise analysis using sequences with a high level of codon bias. Unfortunately, current databases do not contain a sufficient number of sequences for many of the genetic codes.

Although our proposed optimal maps did not provide the best results for all scenarios, the correlation coefficient always exceeded 0.9 and the maximal percentage deviation was kept under 8%. The value of percentage deviation may seem quite high, but the translation itself from codons to amino acids causes loss of signal resolution as the value range of amino acid signal is one-third of the codon signal and the range reduction is not linear because of the genetic code degeneration.

In addition to the verification of proposed optimal numerical maps for particular genetic codes, the globally optimal map was verified. Such a map can be applied for general use in analyses without a specified genetic code or not permitting a change of settings. The globally optimal map was the best possible solution for 15 of the 24 genetic codes based on our optimization criterion and for eight of the 13 sets of real sequences. Moreover, our globally optimal map was, for 10 of 13 real datasets, better than the original optimal map.

The worst result from all variants of numerical maps suffered from a percentage deviation of over 20%, which is more than two times worse than the worst result of the proposed optimal maps. In such cases, it is not possible to differentiate between the deviation caused by translation and real mutations in sequences. Conclusively, usage of the optimal numerical map is important and the random assignment of numbers to nucleotides is not reliable.

An example of phylogenetic analysis based on comparison of signals was conducted to demonstrate the effect of usage of different variants of the numerical maps. Three phylogenetic trees were constructed from the coding sequences of mammalian HBB genes. Only the tree based on the proposed optimal numerical map had comparable topology with the reference taxonomy. As the analysis demonstrates, even such a simple task is highly dependent on the utilized numerical map, while poor results are obtained for non-optimal maps.

## Competing Interests

The authors declare that they have no competing interests.

## Declarations of interest

None.
